# The Algorithm and Structure for Digital Normalized Cross-Correlation by Using First-Order Moment †

**DOI:** 10.3390/s20051353

**Published:** 2020-03-01

**Authors:** Chao Pan, Zhicheng Lv, Xia Hua, Hongyan Li

**Affiliations:** 1 School of Information and Communication Engineering, Hubei University of Economics, Wuhan 430205, China; PanChao@hbue.edu.cn (C.P.); lion112@139.com (Z.L.); hongyanli2000@126.com (H.L.); 2 School of Electrical and Information Engineering, Wuhan Institute of Technology, Wuhan 430205, China

**Keywords:** normalized cross-correlation, fast algorithm, first-order moment, systolic array, multiplication complexity

## Abstract

Normalized cross-correlation is an important mathematical tool in digital signal processing. This paper presents a new algorithm and its systolic structure for digital normalized cross-correlation, based on the statistical characteristic of inner-product. We first introduce a relationship between the inner-product in cross-correlation and a first-order moment. Then digital normalized cross-correlation is transformed into a new calculation formula that mainly includes a first-order moment. Finally, by using a fast algorithm for first-order moment, we can compute the first-order moment in this new formula rapidly, and thus develop a fast algorithm for normalized cross-correlation, which contributes to that arbitrary-length digital normalized cross-correlation being performed by a simple procedure and less multiplications. Furthermore, as the algorithm for the first-order moment can be implemented by systolic structure, we design a systolic array for normalized cross-correlation with a seldom multiplier, in order for its fast hardware implementation. The proposed algorithm and systolic array are also improved for reducing their addition complexity. The comparisons with some algorithms and structures have shown the performance of the proposed method.

## 1. Introduction

Normalized cross-correlation (NCC) is an important mathematical tool in signal and image processing for feature matching, similarity analysis, motion tracking, object recognition, and so on [1,2,3]. In order to improve its real-time and efficient performance, digital NCC has been suggested to be implemented by some fast algorithms and hardware structures, due to its high computational complexity [4,5].

Nowadays, since correlation and convolution have similar computation structures, there are mainly three kinds of fast convolution algorithms can be applied for fast NCC [6,7]: (1) the Fast Fourier Transform (FFT)-based algorithm, (2) the polynomial-based algorithm, (3) the decomposition algorithm. However, to our knowledge, each of these algorithms has its applicable limitations. The FFT-based algorithm is not well-suited to the discrete domain. Plus, it involves with complex multiplications [8,9]. Both the polynomial-based algorithm and the decomposition algorithm require complex computational structures, and they often lack commonality for arbitrary-length correlations [10,11]. 

Furthermore, some special algorithms for fast NCC have been presented [12,13]. The fast cross-correlation of binary sequences can be extended to other types of NCC sequences [14]. The estimation algorithm derives the scaling factor between the signal and the kernel, so it computes NCC using only additions at the cost of small noise [15]. Several methods have been used to assist NCC for reducing its searching and computing times in image matching, such as the pyramid method [3,7]. In addition, many parallel algorithms of the inner-product have been published that can perform fast cross-correlation for NCC [16,17], where the Distributed Arithmetic (DA) with look-up table has not multiplication, but needs much Read-Only Memory (ROM) [18].

To hardware implementation of fast NCC, Very-Large-Scale Integration (VLSI) circuits have been applied, where systolic structures are popular due to their regularity and modularity [19,20,21]. The integration of the systolic array and the DA technique lead to more efficient VLSI implementation of cross-correlation, although they use many ROMs and address decoders [22,23]. The Residue Number System-based DA can reduce ROMs and enhance throughput, while extra encoding processes in the residue domain are necessary [24].

In this paper, we present a new algorithm and structure to implement digital NCC with a simple and fast procedure. It is a breakthrough that an NCC formula expressed in terms of a first-order moment is designed according to the relationship between the inner-product and the first-order moment, so the computational complexity of NCC is transformed into that of a first-order moment. For performing an arbitrary-length digital NCC, our algorithm would first establish the NCC formula based on a first-order moment for correlation sequences, and then introduce a fast algorithm without multiplication from [25,26] to compute this first-order moment in the new NCC formula rapidly. For the hardware implementation of NCC, we develop a simple and scalable systolic array derived from the proposed algorithm, due to the fact that the fast algorithm for the first-order moment is easily performed by systolic structure [27]. The proposed algorithm and systolic array are also improved to reduce their addition complexity, according to an even-odd relationship in the computation of the first-order moment.

The rest of the paper is organized as follows. Section 2 establishes the NCC formula based on a first-order moment. Section 3 introduces a fast algorithm and its systolic implementation for first-order moment. Section 4 and Section 5 discuss the fast algorithm and the systolic array inspired by Section 3 to perform the NCC formula in Section 2 rapidly. Comparison and analysis are presented in Section 6 to demonstrate the feasibility of the proposed algorithm and structure. Finally, Section 7 gives the conclusion.

## 2. Normalized Cross-Correlation Based on First-Order Moment

Being the most complex operation in NCC, the inner-product of two correlation sequences would be transformed into a first-order moment for decreasing computational complexity in fast NCCs. To do this, let us assume two *N*-point digital sequences { *f*(*i*) } and { *g*(*i*) }, where { *f*(*i*) } is an arbitrary input sequence, and { *g*(*i*) } is the fixed correlation kernel with the value range *g*(*i*)∈{ 0, 1, 2, …, *L* }. This section establishes an NCC formula for these two sequences that mainly includes a first-order and a zero-order moment. The aim is to replace the complex computation of cross-correlation in NCC with an easy computation of a first-order moment.

### 2.1. Cross-Correlation

Cross-correlation is an inner-product between two digital sequences. It is defined as

(1) $$c(n)=f(n)\circ g(n)=\sum_{i=0}^{N-1}f(n+i)g(i)$$

Using mathematical transformation, this Equation (1) could be transformed into a first-order moment by means of the statistical characteristics of the inner-product operation. To do this, we define some subsets *S_k_* (*k* = 0, 1, 2, …, *L*) that divide the index set *i*∈{0, 1, …, *N* − 1} into *L* subsets, depending on the max value in the correlation kernel { *g*(*i*) }. Specifically,

(2) $$S_{k}=\left\{\;i\;|\;g(i)=k,\;i\in \left\{0,\;1,\;2,\;\cdots ,\;N-1\right\}\;\right\}$$

where *k* = 0, 1, 2, …, *L*. In other words, *S_k_* is a set of indices *i* that corresponds to *g*(*i*) = *k* in actual. Then a new (*L* + 1)-point sequence { *a_k_*(*n*) } is defined by subsets *S_k_* [28], which is

(3) $$a_{k}(n)\;=\{\begin{array}{cc} \sum_{i\in S_{k}}f(n+i) & \mathrm{where}\;S_{k}\neq \Phi \\ 0 & \mathrm{otherwise} \end{array},$$

where *k* = 0, 1, 2, …, *L*, and “Φ” denotes an empty set.

The *a_k_*(*n*) could be acted as the sum of elements in the sequence { *f*(*n* + *i*) } while the parameter *i* corresponds to *g*(*i*) = *k*. The computation of the { *a_k_*(*n*) } is actually a statistics procedure for counting how much *k* would be accumulated in the computation of the *c*(*n*). Therefore, the relationship between { *f*(*n* + *i*) } and { *a_k_*(*n*) } can be described as:

(4a) $$\sum_{i=0}^{N-1}f(n+i)=\sum_{k=0}^{L}a_{k}(n),$$

(4b) $$\sum_{i=0}^{N-1}f(n+i)g(i)=\sum_{k=0}^{L}a_{k}(n)k=\sum_{k=1}^{L}a_{k}(n)k$$

It is obvious that $\sum_{k=1}^{L}a_{k}(n)$ in Equation (4a) is a zero-order moment of { *a_k_*(*n*) }, and $\sum_{k=1}^{L}a_{k}(n)k$ in Equation (4b) is a first-order moment of { *a_k_*(*n*) }. As a result, the Equation (1) can be transformed into:

(5) $$c(n)=\sum_{k=1}^{L}a_{k}(n)k$$

From Equation (5), we obtain a new calculation formula for cross-correlation based on a first-order moment.

### 2.2. Normalized Cross-Correlation

Normalized cross-correlation is more complex than cross-correlation, because it includes an inner-product between two difference sequences from { *f*(*i*) }, { *g*(*i*) } and their mean value. It is defined as

(6) $$\rho (n)=\frac{\sum_{i=0}^{N-1}\left[f(n+i)-\bar{f}(n)][g(i)-\bar{g}\right]}{{\left\{\sum_{i=0}^{N-1}{\left[f(n+i)-\bar{f}(n)\right]}^{2}\sum_{i=0}^{N-1}{\left[g(i)-\bar{g}\right]}^{2}\right\}}^{\frac{1}{2}}},$$

where $\bar{f}(n)=\frac{1}{N}\sum_{i=0}^{N-1}f(n+i)$ and $\bar{g}=\frac{1}{N}\sum_{i=0}^{N-1}g(i)$.

This Equation (6) can be rewritten as

(7) $$\begin{array}{ll} \rho (n) & =\frac{\sum_{i=0}^{N-1}f(n+i)g(i)-\bar{g}\sum_{i=0}^{N-1}f(n+i)-\bar{f}(n)\sum_{i=0}^{N-1}g(i)+N[\bar{f}(n)\bar{g}]}{{\left\{\sum_{i=0}^{N-1}\left[f{\left(n+i\right)}^{2}-2f(n+i)\bar{f}(n)+\bar{f}{\left(n\right)}^{2}\right]\sum_{i=0}^{N-1}{\left[g(i)-\bar{g}\right]}^{2}\right\}}^{\frac{1}{2}}}\\ & =\frac{\sum_{i=0}^{N-1}f(n+i)g(i)-\frac{1}{N}\sum_{i=0}^{N-1}f(n+i)\sum_{i=0}^{N-1}g(i)}{{\left\{\{\sum_{i=0}^{N-1}f{\left(n+i\right)}^{2}-\frac{1}{N}{\left[\sum_{i=0}^{N-1}f(n+i)\right]}^{2}\}\sum_{i=0}^{N-1}{\left[g(i)-\bar{g}\right]}^{2}\right\}}^{\frac{1}{2}}}\; \end{array}$$

If we set

(8) $$b(n)=\sum_{i=0}^{N-1}{\left[f(n+i)\right]}^{2}$$

and substitute Equations (4a), (4b) and (8) into Equation (7), the NCC expressed by Equation (6) can be converted to

(9) $$\rho (n)=\frac{\sum_{k=1}^{L}a_{k}(n)k-\bar{g}\sum_{k=0}^{L}a_{k}(n)}{{\left\{\{b(n)-\frac{1}{N}{\left[\sum_{k=0}^{L}a_{k}(n)\right]}^{2}\}\sum_{i=0}^{N-1}{\left[g(i)-\bar{g}\right]}^{2}\right\}}^{\frac{1}{2}}}.$$

From Equation (9), we develop a new calculation formula for NCC based on a first-order moment $\sum a_{k}(n)k$ and a zero-order moment $\sum a_{k}(n)$. It is obvious that the computation complexity of this NCC formula depends heavily upon the complexity of $\sum a_{k}(n)k$ and *b*(*n*). Therefore, for a fast implementation of Equation (9), we introduce a fast algorithm and structure for $\sum a_{k}(n)k$ in Section 3, and an optimization method for *b*(*n*) in Section 4.1.

## 3. The Fast Algorithm and Systolic Array for First-Order Moment

Liu et al. presented an algorithm and its systolic array for first-order moment in [25,26,27]. Their method is suitable to compute the first-order and the zero-order moment in Equation (4) rapidly. In this section, we introduce this algorithm and systolic array that aims to implement fast NCC by using Equation (9). In addition, because the introduced algorithm and array request many additions as the result of removing all multiplications, we also improve them in order for lower addition complexity.

### 3.1. The Fast Algorithm for First-Order Moment

**Figure 1:**
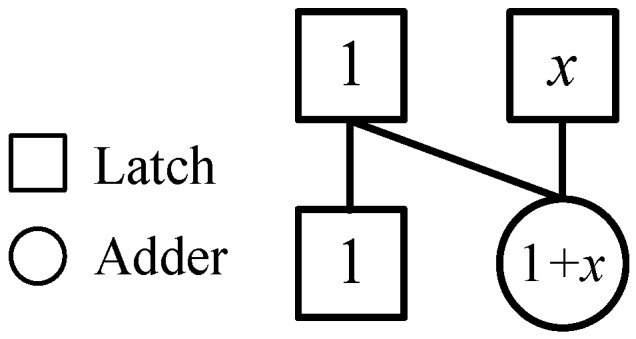
The 1-network.

According to [25], we illustrate a simple 1-network shown in Figure 1 that represents a map of transforming the two-dimensional vector (1, *x*) into the vector (1, (1 + *x*)). This map is denoted by *F* that is

F(1, x)=(1, (1+x)).

Some characteristic equations obtained from *F* are

(10)  F(a, ax)=(a, a(1+x)),  F(a+b, a+b)=F(a, a)+F(b, b)

Also,

$$\;F^{2}(1,\;x)=F(F(1,\;x))=F(1,\;(1+x))=(1,\;2+x)$$

and by induction

$$\;F^{L-1}(1,\;x)=F(\ldots F\ldots F(1,\;x))=(1,\;(L-1+x)).$$

Hence, we have

$$\;F^{L-1}(1,\;1)=F(\ldots F\ldots F(1,\;1))=(1,\;L),$$

(11) $$\;F^{L-1}(a,\;a)=(a,\;La).$$

To compute first-order moment by this 1-network, let

$$\;a_{k}=(\;a_{k}(n),\;a_{k}(n)\;)\;\;(k=1,\;2,\;\ldots ,L),$$

so, Equations (10) and (11) are yielded by

$$\begin{array}{l} F(F(a_{k})+a_{k-1})=F(F(a_{k}))+F(a_{k-1})=F^{2}(a_{k})+F(a_{k-1})\\ =(\;a_{k}(n)+a_{k-1}(n),\;3a_{k}(n)+2a_{k-1}(n)\;) \end{array}$$

Generally, the above equation is expanded into

(12) $$\begin{array}{l} F(F\ldots F(F(F(a_{L})+a_{L-1})+\ldots )+a_{2})+a_{1}=F^{L-1}(a_{L})+\ldots +F^{2}(a_{3})+F(a_{2})+a_{1}\\ =(\sum_{k=1}^{L}a_{k}(n),\sum_{k=1}^{L}a_{k}(n)k) \end{array}.$$

**Figure 2:**
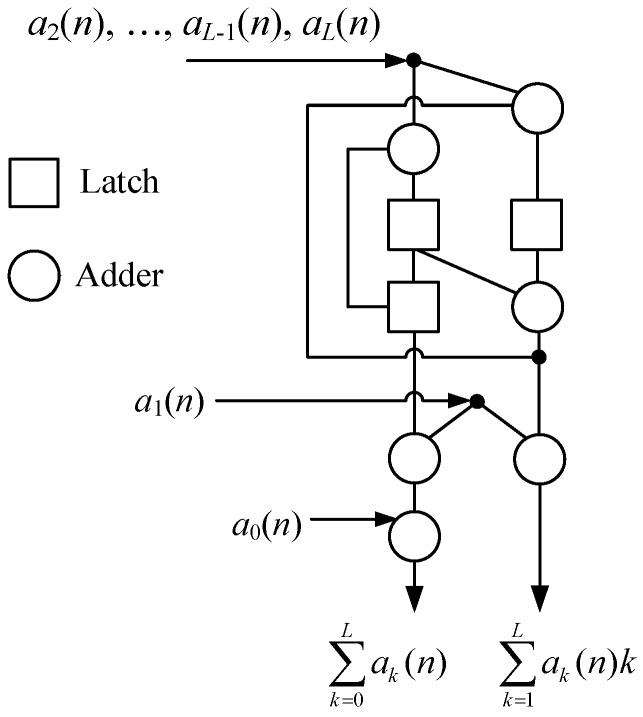
The computational structure for first-order moment.

From Equation (12), $\sum a_{k}(n)$ in Equation (4a) and $\sum a_{k}(n)k$ in Equation (4b) can both be obtained from an iterative implementation of the map *F*. This computational flow uses the (*L* − 1) recursive process of map *F* that includes 3*L* additions and 0 multiplications [26]. Therefore, the fast algorithm for first-order moment by Equation (12) can be described in Algorithm 1 as a subroutine Moment [29]. Its computational structure is also shown in Figure 2, which is an iterative structure of a 1-network with six adders and three latches. Its total addition number to compute *N*-point first-order moments $\sum a_{k}(n)k$ (*n* = 0, 1, …, *N* − 1) is 3*NL*.
**Algorithm 1 Moment** (*a_L_*(*n*), *a_L_*_− 1_(*n*), …, *a*_0_(*n*)) Define the array **a** with two elements Initial **a**
← ( *a_L_*(*n*), *a_L_*(*n*) ) **for** each *k*
∈ [2, *L*]  do  // Equation (12)  **a**[1] ←
**a**[1] + **a**[0]   // 1-network *F*(**a**)  **a**[1] ←
**a**[1] + *a_L-k+_*_1_(*n*)  **a**[0] ←
**a**[0] + *a_L-k+_*_1_(*n*) **end for** **a**[0] ←
**a**[0] + *a*_0_(*n*) **return a**

### 3.2. The Systolic Array for First-Order Moment

**Figure 3:**
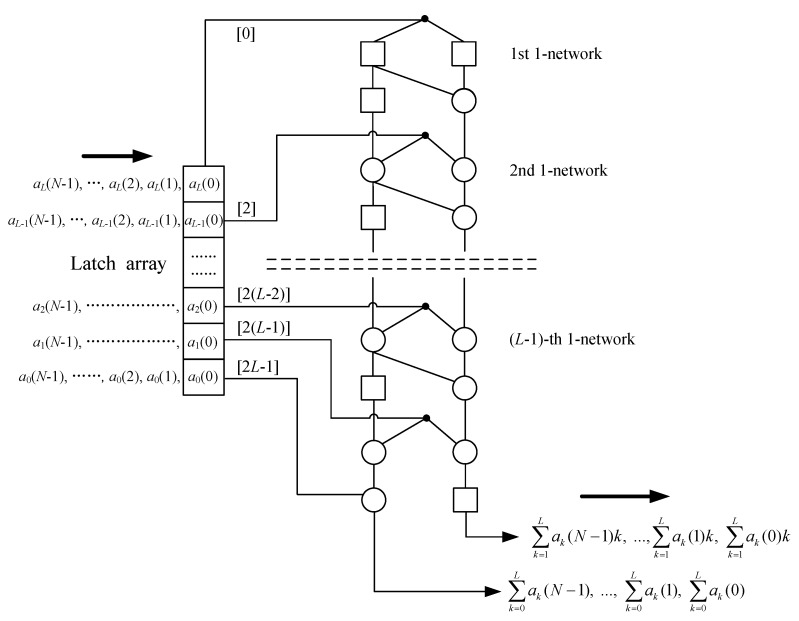
The systolic array for first-order moment.

The Equation (12) can be implemented by a systolic array for continuously generating a set of $\sum a_{k}(n)$ and $\sum a_{k}(n)k$ in parallel [27]. This systolic array is shown in Figure 3, which is actually a serial arrangement of (*L* − 1) 1-networks extended from Figure 2. It uses 3*L* − 2 adders, *L* + 2 latch, and 0 multiplier. In each clock cycle, we should input a sequence { *a_k_*(*n*) } into this systolic array and get a $(\sum a_{k}(n),\;\sum a_{k}(n)k)$.

Especially, to keep an operation synchronization for this parallel structure, the (*L* − 1)-point *a_k_*(*n*) (*k* = 2, …, *L*) should be input into the (*L* − 1) 1-networks respectively rather than simultaneously. Generally, a single *a_k_*(*n*) (*k* > 0) is input into the (*L* − *k*)-th 1-network with a latency *n* + 2 (*L* – 1 − *k*) clock cycle. Hence, in Figure 3, we use the extra latch array to generate latency for *a_k_*(*n*) before it is input into the corresponding 1-network. The number of latch array and latency time is shown in the note “[ ]”, which leads to the occurrence that different *a_k_*(*n*) are input into the different 1-networks at regular intervals. As a result, the total execution time of this systolic array to compute *N*-point $\sum a_{k}(n)k$ (*n* = 0, 1, …, *N* − 1) is that

2L−1+1+N−1=2L+N−1

clock cycles.

### 3.3. The Improvement of the Fast Algorithm and Systolic Array for First-Order Moment

The algorithm in Section 3.1 requires many additions that are computationally expensive when *N* is larger. In order to reduce its addition number, this algorithm is improved by means of an even-odd relationship that divides the first-moment of sequence { *a_k_*(*n*) } into two smaller moments. This even–odd relationship is illustrated as:

(13a) $$\sum_{k=0}^{L}a_{k}(n)=\sum_{k=1}^{L/2}\left[a_{2k-1}(n)+a_{2k}(n)\right]+a_{0}(n),$$

(13b) $$\sum_{k=1}^{L}a_{k}(n)k=\sum_{k=1}^{L/2}a_{2k-1}(n)\cdot (2k-1)+\sum_{k=1}^{L/2}a_{2k}(n)\cdot 2k=2\sum_{k=1}^{L/2}\left[a_{2k-1}(n)+a_{2k}(n)\right]k-\sum_{k=1}^{L/2}a_{2k-1}(n).$$

**Figure 4:**
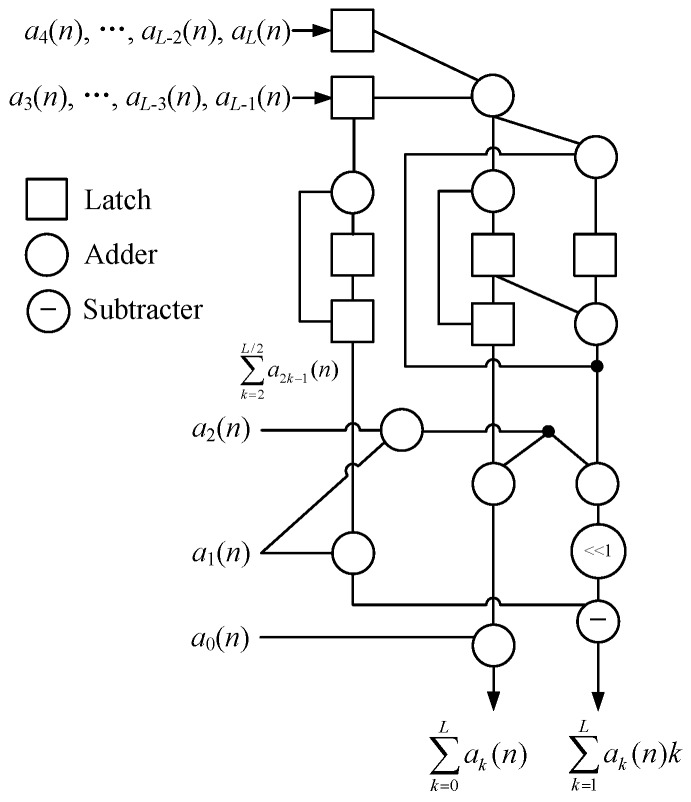
The improved computational structure for first-order moment.

According to Equation (13), the fast algorithm described by Figure 2 can be improved to the new structure shown in Figure 4. This improved algorithm firstly adds *L*/2 additions to obtain the sequence $\{\;a_{2k-1}(n)+a_{2k}(n)\;\}$ as well as *L*/2 − 1 addition to accumulate $\sum a_{2k-1}(n)$. Then each $a_{2k-1}(n)+a_{2k}(n)\;$ is input into map *F* successively for performing *L*/2 − 1 iterations. Finally, a left-shift operation and 1 subtraction are applied to generate $\sum a_{k}(n)k$. The improved algorithm requires 5*L*/2 − 1 additions that are superior to Figure 2, even though its structure is more complex at the cost of decreasing *L*/2 additions. Although the sequence $\{\;a_{2k-1}(n)+a_{2k}(n)\;\}$ could be continually divided by the even-odd relationship for further reducing additions, the fast algorithm’s structure would become very complex and unworthy. 

**Figure 5:**
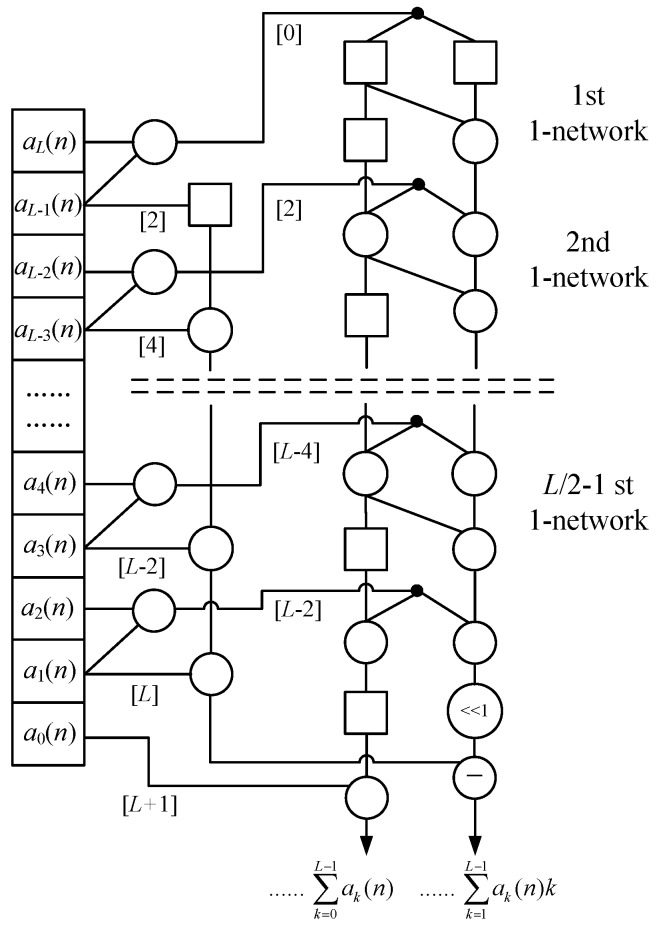
The improved systolic array for first-order moment.

Similarly, the systolic array in Figure 3 can be improved to the structure shown in Figure 5. This improved systolic array is a serial arrangement of the *L*/2 − 1 1-networks extended from Figure 4. It requires 5*L*/2 − 3 adders and *L*/2 + 3 latches that are superior to Figure 3, even though its structure is more complex. As a result, the total execution time of this systolic array to compute *N*-point $\sum a_{k}(n)k$ (*n* = 0, 1, …, *N* − 1) is decreased to

L+1+1+N−1=N+L+1

clock cycles.

## 4. The Fast Algorithm for Normalized Cross-Correlation 

We apply the improved fast algorithm in Section 3.3 to compute the first-order and the zero-order moments in Equation (9). Thus, the fast algorithm for NCC is presented that can remove most of its multiplications. At first, some optimization methods are introduced in Section 4.1 to further reduce its additions.

### 4.1. The Optimization Methods

As the sequence { *g*(*i*) } is a fixed correlation kernel in general, both $\bar{g}=\sum g(i)/N$ and $\sum {\left[g(i)-\bar{g}\right]}^{2}$ in Equation (9) could be pre-computed and reused for avoiding their repeated computations [30].

Although *b*(*n*) in Equation (8) involves many additions and complex squares, it could also be computed by a simple function with the previous *b*(*n* − 1), where

(14) $$\begin{array}{ll} b(n) & =\sum_{i=0}^{N-1}f{\left(n-1+i\right)}^{2}+[f{\left(n+N-1\right)}^{2}-f{\left(n-1\right)}^{2}]\\ & =b(n-1)+[f(n+N-1)+f(n-1)][f(n+N-1)-f(n-1)] \end{array}$$

We only need to directly compute the first *b*(0) by *N* multiplication and *N* − 1 additions, where the square is performed by multiplication. Then, the following *b*(*n*) (*n* = 1, 2, …, *N* − 1) would be obtained from Equation (14) by only 1 multiplication, 2 additions and 1 subtraction.

### 4.2. The Step of the Fast Algorithm for NCC

The proposed fast algorithm for NCC would include five steps:

**Step 1**  Initializing all *a_k_*(*n*) = 0 (*k* = 0, 1, …, *L*), where *a*_0_(*n*) is indispensable for $\sum a_{k}(n)$.
**Step 2**  Implementing Equation (3) to acquire the sequence { *a_k_*(*n*) } using *N* addition.
**Step 3**  Computing $\sum a_{k}(n)$, $\sum a_{k}(n)k$ by Equation (13) and Figure 4 with 5*L*/2 − 1additions.
**Step 4**  Computing *b*(*n*) by Equation (14) with 1 multiplication, 2 additions and 1 subtraction.
**Step 5**  Inputting $\sum a_{k}(n)$, $\sum a_{k}(n)k$ and *b*(*n*) into Equation (9) for a NCC *ρ*(*n*), which need 2 subtractions, 4 multiplications, 1 division and 1 square root calculation.

The computational flow of this algorithm is illustrated in Algorithm 2. It includes *N* + 5*L*/2 + 1 additions, 3 subtractions and 5 multiplications per output an NCC *ρ*(*n*). Therefore, to compute *N*-point NCC, it requires *N* − 1 + *N* (*N* + 5*L*/2 + 1) − 2 = *N* (*N* + 5*L*/2 + 2) − 3 additions, and only *N* + *N* − 1 + 4*N* = 6*N* − 1 multiplications.
**Algorithm 2 Computing NCC** ( *n*, *f*, *g*, *b*(*n*-1) ) **for** each *a_k_* in the sequence { *a_k_* }: *a_k_*
← 0  **for** each *i*
∈ [0, *N*-1] do       // Equation (3)  *k*
←
*g*(*i*)   *a_k_*
←*a_k_* + *f*(*n* + *i*)  **end for** **for** each *k*
∈ [1, *L/2*] do       // Equation (13a)
   *s*
←
*s + a*_2*k*−1_

   *a_k_*
←
*a*_2*k*−1_ + *a*_2*k*_
 **end for**
 **a**
←
**Moment** ( *a_L_*_/2_, *a_L_*_/2−1_, …, *a*_2_, *a*_1_, *a*_0_)   // Algorithm 1 
 **a**[1] ←
**a**[1] << 1 – *s*           // Equation (13b)
 Compute *b*(*n*) by *b*(*n*-1), *f*(*n* + *N* − 1) and *f*(*n* − 1) // Equation (14)
 Compute *ρ*(*n*) by **a**[0], **a**[1] and *b*(*n*)      // Equation (9) 
 **return**
*ρ*(*n*)

## 5. The Systolic Array for Normalized Cross-Correlation 

**Figure 6:**
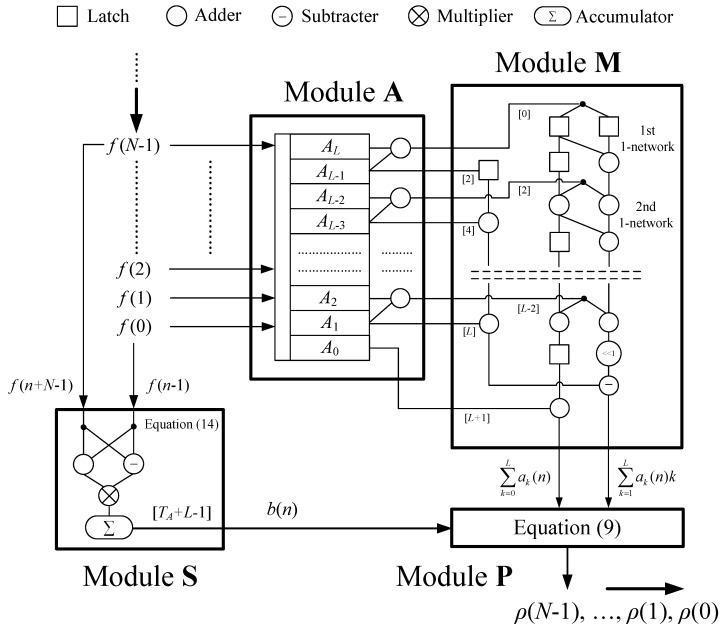
The systolic array for fast normalized cross-correlations (NCCs).

We apply the improved systolic array in Figure 5 to design a hardware structure for fast NCC in parallel. Figure 6 shows this systolic structure that mainly includes three parts: the module **A** to compute $\{\;a_{2k-1}(n)+a_{2k}(n)\;\}$, the module **M** to compute the first-order and zero-order moment of { *a_k_*(*n*) }, and the module **S** to compute *b*(*n*). In each cycle, we simultaneously input *N*-point *f*(*n* + *i*) into this systolic array and get an NCC result *ρ*(*n*). At first, since the direct computation for $\{\;a_{2k-1}(n)+a_{2k}(n)\;\}$ needs many adders, a simplified structure for the module **A** is discussed in Section 5.1.

### 5.1. The Module **A**

The module **A** is to acquire an *L*/2-point sequence $\{\;a_{2k-1}(n)+a_{2k}(n)\;\}$ according to Equations (3) and (13) in every clock cycle. It includes *L* + 1 sub-modules *A_k_* (*k* = 0, 1, 2, …, *L*) that firstly count { *f*(*n* + *i*) } to generate corresponding { *a_k_*(*n*) }, and then sum up the two adjacent *a_k_*(*n*) to obtain $\left\{\;a_{2k-1}(n)+a_{2k}(n)\;\right\}$. We assume the execution time of the module **A** is *T_A_* clock cycles. The *N*-point *f*(*n* + *i*) should be inputted into the sub-modules { *A_k_* } in a gradual way. 

**Figure 7:**
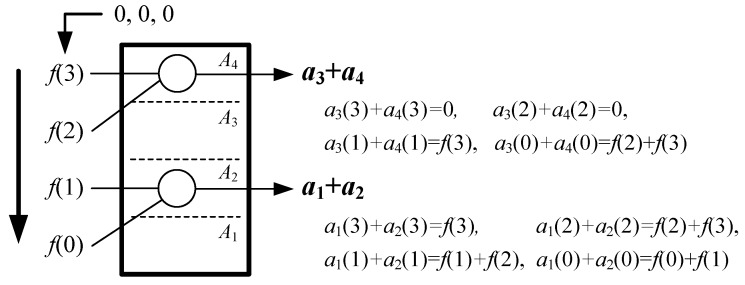
The module **A** for { *g*(*i*) } = {1, 2, 3, 4}.

**Figure 8:**
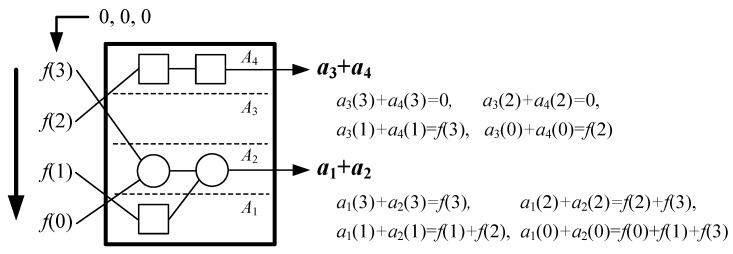
The module **A** for { *g*(*i*) } = {2, 1, 4, 2}.

**Figure 9:**
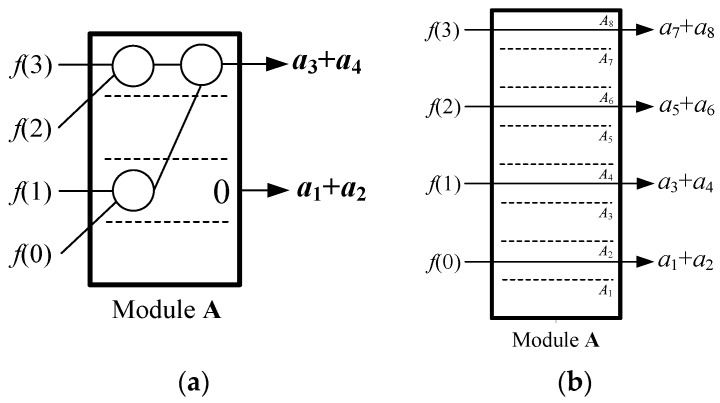
The module **A** using different adders: (**a**) { *g*(*i*) } = {4, 4, 4, 4}; (**b**) { *g*(*i*) } = {2, 4, 6, 8}.

Since the correlation kernel { *g*(*i*) } is so invariable that the computational strategy for Equations (3) and (13) are known in advance, we could simplify the structure of *A_k_* for less adder and data transfer. For example, for *N* = 4, *L* = 4 and { *g*(*i*) } = { 1, 2, 3, 4 }, the module **A** could be simplified as shown in Figure 7 with 2 adder and *T_A_* = 1. However, for *N* = 4, *L* = 4 and { *g*(*i*) } = { 2, 1, 4, 2 }, the module A would be re-designed as shown in Figure 8 with 2 adder, 3 latches and *T_A_* = log_2_4 = 2. Therefore, the structure of the module **A** should be not fixed, but changed with different sequences { *g*(*i*) } to reduce its hardware complexity. We also show the module **A** using maximum adders when { *g*(*i*) } = { 4, 4, 4, 4 } in Figure 9a, and the module **A** using 0 adders when { *g*(*i*) } = { 2, 4, 6, 8 } in Figure 9b. From Figure 7, Figure 8 and Figure 9, it can be obtained the adder number of the module **A** is from 0 to *N* − 1, and the latency *T_A_* is from 0 to log_2_*N*.

### 5.2. The Model **P**

The Model **P** is to implement Equation (9) with 4 multipliers, 1 divider and 1 square root extractor. It receives a $\sum a_{k}(n)k$ and a *b*(*n*), and output a corresponding *ρ*(*n*) in each cycle. Some fast methods can be applied for the square root operation. In addition, the fixed $\bar{g}$ and $\sum {\left[g(i)-\bar{g}\right]}^{2}$ are saved in advance against repeated computation.

### 5.3. The Systolic Array

The systolic array in Figure 6 uses various modules to perform Equations (3), (9), (13) and (14), respectively, for NCC. Some latches are indispensable to connect these modules for assuring their mutual and parallel operation. The latch number has been shown in the note “[ ]”. The module M from Figure 5 is to compute first-order moments and zero-order moments based on Equation (13). The module **S** implements Equation (14) and generates *b*(*n*) by 1 multiplier, 1 accumulator and 1 subtractor. Finally, the module **P** generates NCC *ρ*(*n*). The systolic array’s total adder number is ranged from 2*L* − 2 to 2*L* + *N* − 3, and its multiplier number is 5.

The initial value of the accumulator in the module **S** is set as *b*(0). In the *n*-th clock cycle, *f* (*n* + *N* − 1) and *f* (*n* − 1) would be input into the module **S** to get *b*(*n*) with three clock cycles. Then *b*(*n*) is output from the module **S** to the module **P** with a latency *T_A_ + L* − 1. The aim is that *b*(*n*), $\sum a_{k}(n)$ and $\sum a_{k}(n)k$ can arrive in the **P** at the same time.

## 6. Comparisons 

The proposed algorithm and systolic structure are compared with some existing methods to verify their effectiveness. These compared methods are also focused on reducing their multiplication numbers.

### 6.1. Algorithm Comparison

Because correlation and convolution can share fast algorithms, we compare the proposed algorithm in Section 4 with some convolution algorithms, as well as a fast NCC algorithm to compute an *N*-point cyclic NCC. The computational complexity of these algorithms are displayed in Table 1, where we set a complex multiplication, which is equivalent to three real multiplications and three real additions, an “AND” operation is equivalent to an addition [31], and a subtraction is also equivalent to an addition.

**Figure 10:**
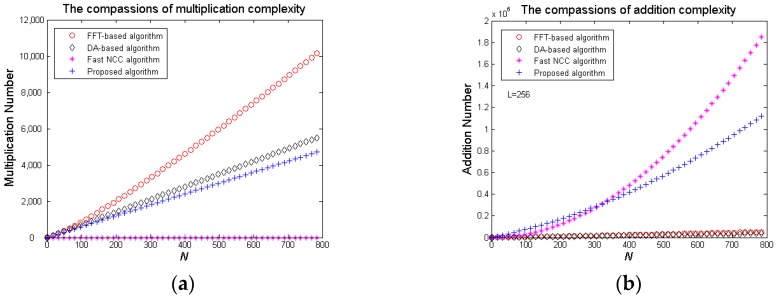
The four algorithm’s multiplication and addition number: (**a**) Multiplication (**b**) Addition.

From Table 1, the multiplication and addition complexity of the FFT-based algorithm are both *O*(*N* log_2_*N*), the DA-based algorithm is the least addition complexity, and the fast NCC algorithm has zero multiplication. The proposed algorithm uses *O*(*N*^2^) additions that are more than the FFT-based and the DA-based algorithm, and *O*(*N*) multiplications that are more than the fast NCC algorithm. However, the FFT-based algorithm needs float addition and multiplication operations that are more complex than integer operations, the DA-based algorithm requires tedious decode address and very large memories, as well as that the fast NCC algorithm is the most addition complexity and not suitable for high-precision matching [15]. Figure 10 shows the four algorithms’ multiplication and addition number increasing along with *N*. It is obviously that the proposed algorithm’s multiplication number is lower than both the FFT-based algorithm’s and the DA-based algorithm’s, and its addition number is lower than the fast NCC algorithm’s when *N* > 320.

**Figure 11:**
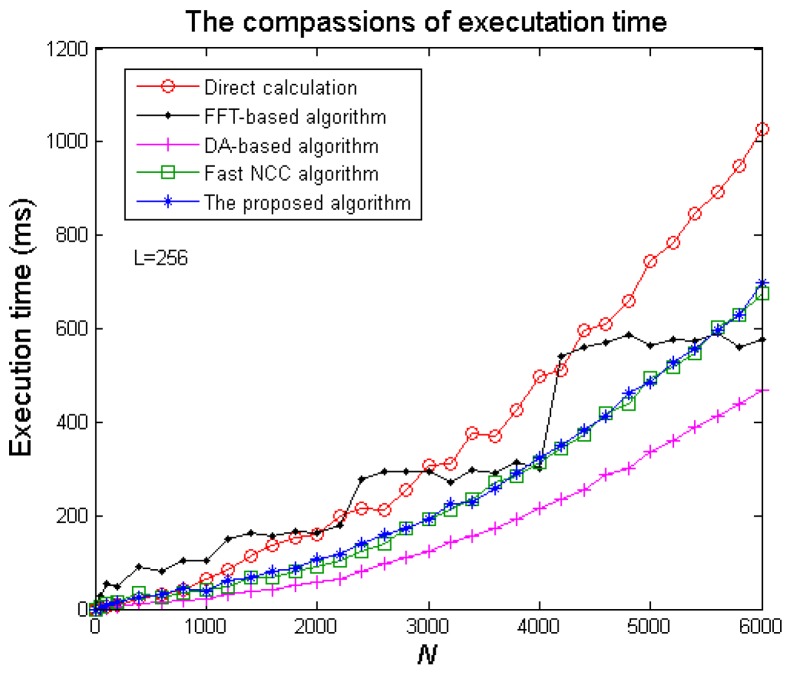
The comparisons of the five algorithm’s execution time (ms).

The wireless sensor and communication is an important application field for the proposed algorithm. Therefore, we compare the execution time of the five algorithms from Table 1 by using a mobile phone with the type “HUAWEI nova 2s (HWI-AL00)” and the operation system “Android 9”. Figure 11 shows these algorithms’ execution time to compute a cyclic NCC by the phone with *N* from 100 to 6000. The growth curve of the FFT-based algorithm’s time is similar to a step curve, in that the length of FFT needs to be extended from *N* to $2^{\left\lceil \log_{2}N\right\rceil }$. Although the DA-based algorithm can use the least time, it needs too much memory to make it worthwhile. From the Figure 11, the proposed algorithm’s execution time is less than the FFT-based algorithm’s when *N* < 5500, and is very close to the fast NCC algorithm, but not involved with noise. 

In addition, it is important that the proposed algorithm has five advantages, as follows:

(1) With less multiplications and memory.
(2) Simple computational structure due to its simple implementation.
(3) Precision and Fit to discrete domain as it uses integer operations [32].
(4) Without limitations on the length of NCC.
(5) Implementation by simple systolic structure.

### 6.2. Structure Comparison

We compare the proposed systolic array in Section 5 with some existing hardware structures. Table 2 shows the hardware complexity of these structures to implement an *N*-point cyclic NCC, where *N* = *PM* (*P* and *M* are two positive integers derived from [33]). Because the proposed array‘s adder number and latency are not fixed, but varied with the sequence { *g*(*i*) }, we only display their value range according to Section 5.1. The execution time of the model **P** is assumed as three clock cycles.

From Table 2, it is an advantage that the proposed systolic structure does not need ROMs, while the other two structures use O(2*^N^*) ROMs that are hardware-expensive when *N* > 16. The structure [22] has minimum latency, but its throughput is more than 1. The structure [33] needs the O(*P*) adder and latency that would increase rapidly with *N*. 

**Figure 12:**
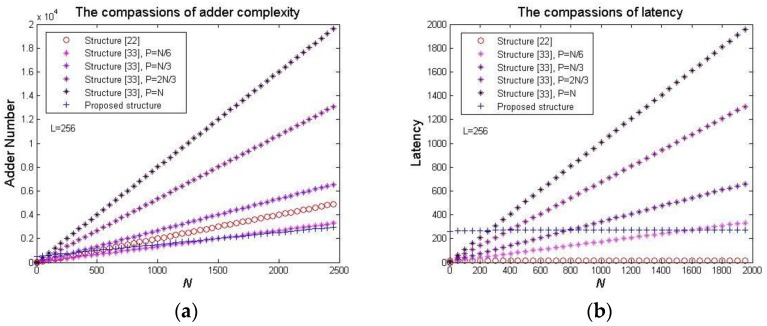
The three structure’s adder number and latency: (**a**) Adder (**b**) Latency.

The proposed structure’s hardware complexity is dependent upon *L*. Furthermore, for long NCCs, or two-dimension NCCs when *N* and *P* are larger than *L*, the adder number of the proposed structure is lower than that of the structure [22], and the latency of the proposed structure is lower than that of the structure [33]. Figure 12 shows the three structures’ adder number and latency increasing along with *N*, where the proposed structure adopts maximum adder and latency to perform comparisons. It is obvious that the proposed structure’s adder number is least when *N* > 1800, and its latency is lower than the structure [33] when *N* > 1500. Therefore, although additional O(*L*) latches are required for data store and transfer, the proposed systolic array could be more efficient in digital signal and image domain where the maximum value of *L* is less than 256 in general [34]. 

## 7. Conclusions

It is suggested that digital NCCs be implemented by efficient algorithms and hardware structures for decreasing their high multiplication complexity [35]. With the assist of fast computation for first-order moment, this paper presents an algorithm and a systolic array for fast NCCs that aim to reduce multiplication as much as possible. To do this, the key is to transform the complex inner-product in the NCC into a simple first-order moment according to the statistical properties of the digital inner-product, and then a new NCC formula based on a first-order moment is established in order for eliminating inner-product operations. As a result, by introducing an algorithm without multiplication into the computation of the first-order moment in NCC, we proposed a fast algorithm for NCC with the advantages of simple implementation, less multiplication, no length limitation, and so on. Especially, as the introduced algorithm for first-order moment requests many additions, we also improved it by means of an even-odd relationship to reduce addition complexity and execution time. It is an advantage that the introduced algorithm for the first-order moment can be implemented by systolic structure, so a systolic array composed of latches and adders is designed for implementing fast NCC in parallel. This systolic array is hardware-efficient due to its parallel operation, simple structures and seldom multiplier. This paper analyzes the computational and the hardware complexity for the proposed algorithm and systolic array, and compares them with some existing methods to prove their efficiency. The proposed algorithm and array could also be applied for digital filter and various transforms [36]. 

There are still many additions in the proposed algorithm and systolic structure. Future studies will focus on further reducing their additions.

## Figures and Tables

**Table 1:** The compassions of computational complexity.

Algorithm	Multiplication	Addition
Direct calculation	*2N* (*N* + 1)	*3N* (*N* + 1)
FFT-based algorithm [8,9]	(3/2) *N*log_2_*N* − (3/2) *N* + 16	(7/2) *N*log_2_*N* − *N/2* + 15
DA-based algorithm [22]	7*N* − 1	(5*N* − 2) log_2_*L* + 8*N* − 1
Fast NCC algorithm [15]	0	3*N* (*N* + 1)
The proposed algorithm	6*N* − 1	*N* (*N* + 5*L*/2 + 5) - 4

**Table 2:** The compassions of hardware complexity.

Complexity	The Proposed Structure	Structure in [22]	Structure in [33]
ROM number	0	[(*N* − 1)/2] [2^3(*N* − 1)/7^]	2*^M^ P* log_2_*L*
Adder Number	2*L* − 2 To 2*L* + *N* − 3	2*N*	(*P* + 1)log_2_*L*
Latency	*L* + 5 To log_2_*N* + *L* + 5	2 log_2_*L*	log_2_*L* + *P*
Throughput	1	*N*/2 log_2_*L*	1
